# Systemic Air Embolism Leading to Cardiorespiratory Arrest Following a CT-Guided Biopsy: A Case Report

**DOI:** 10.7759/cureus.79535

**Published:** 2025-02-23

**Authors:** Daniel Aparício, Miguel L Mendes, Rita Almeida, Carla Eira, Pedro Patrão, Ana Albuquerque

**Affiliations:** 1 Internal Medicine, Unidade Local de Saúde Viseu Dão-Lafões, Viseu, PRT; 2 Internal Medicine, Centro Hospitalar Tondela-Viseu, Viseu, PRT; 3 Critical Care Medicine, Unidade Local de Saúde Viseu Dão-Lafões, Viseu, PRT; 4 Radiology, Unidade Local de Saúde Viseu Dão-Lafões, Viseu, PRT

**Keywords:** air embolism, cardiac arrest, left ventricular air embolism, pulmonary disease, transthoracic guided lung biopsy

## Abstract

CT-guided transthoracic lung biopsy is a commonly performed procedure for diagnosing pulmonary lesions. While generally safe, it has known risks. Pneumothorax, pulmonary hemorrhage, and hemoptysis are among the most frequent complications. Although rare, serious complications such as air embolism can occur, and while uncommon, it is a potentially life-threatening condition that may result from this procedure. It can lead to acute ischemic stroke or acute myocardial infarction, which can be fatal. Here, we describe a case of a 72-year-old man with a right lower lobe pulmonary nodule who had a cardiorespiratory arrest due to air embolism due to a CT-guided biopsy of the pulmonary nodule found on a previous CT scan of the chest. The patient was successfully resuscitated and intubated for mechanical ventilation and admitted to an intensive care unit. He was later transferred to the pulmonology unit and discharged home in stable condition.

## Introduction

CT-guided transthoracic lung biopsy is a commonly performed procedure for diagnosing pulmonary lesions. While generally safe, it has known risks. Pneumothorax, pulmonary hemorrhage, and hemoptysis are among the most frequent complications. Although rare, there are other serious complications already reported in other previous case reports [1], such as air embolism, with reported incidence rates ranging from 0.02% to 0.07%, and sometimes these complications are not detected immediately after the procedure [2,3]. There are some potential risk factors for systemic air embolism as a complication of this procedure, including parenchymal hemorrhage during the procedure, lower lobe lesions, and the use of larger biopsy needles [4-6].

Cardiac arrest at the time of the incident is the primary risk factor for short-term mortality, likely reflecting the large volume of gas that entered the circulation [7].

## Case presentation

We describe a case of a 72-year-old man with a right lower lobe pulmonary nodule who had a cardiorespiratory arrest due to air embolism due to a CT-guided biopsy of the pulmonary nodule. This patient was submitted to a programmed CT-guided biopsy to study a pulmonary nodule found on a previous CT scan of the chest regarding long-time weight loss, cough, and dyspnea. He was medicated only with inhaled umeclidinium bromide/vilanterol and had a smoking exposure of 60 pack-years.

In the immediate post-procedure, the patient experienced a cardiorespiratory arrest. Spontaneous circulation was restored after four minutes of basic life support (BLS). Subsequently, the patient suffered two additional cardiac arrests, both presenting as pulseless electrical activity (PEA), a non-shockable rhythm. Recovery was achieved following the administration of a single dose of epinephrine and two cycles of advanced life support (ALS) (2+2 minutes).

**Figure 1:**
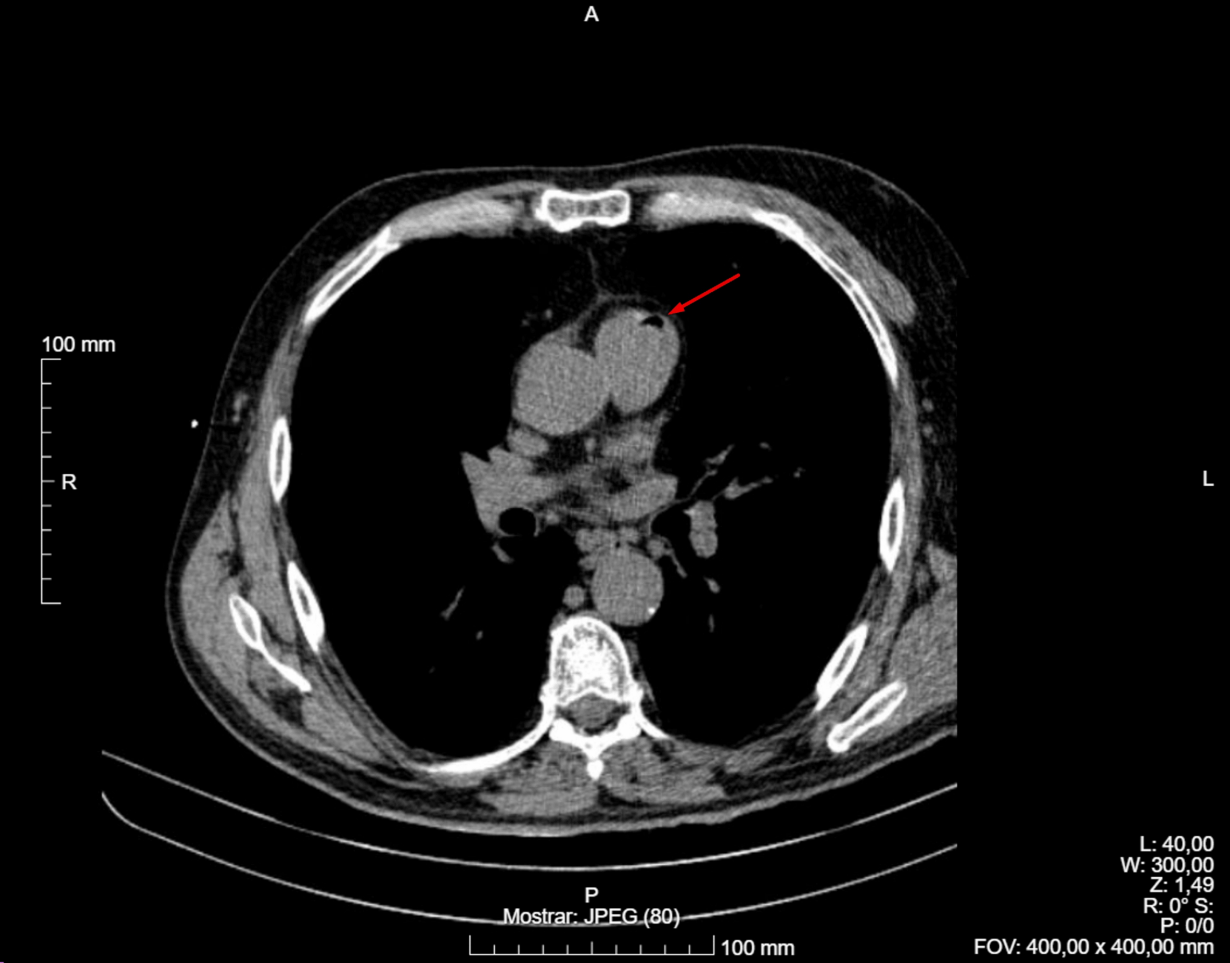
Control CT scan showing air embolism in the left ventricle.

**Figure 2:**
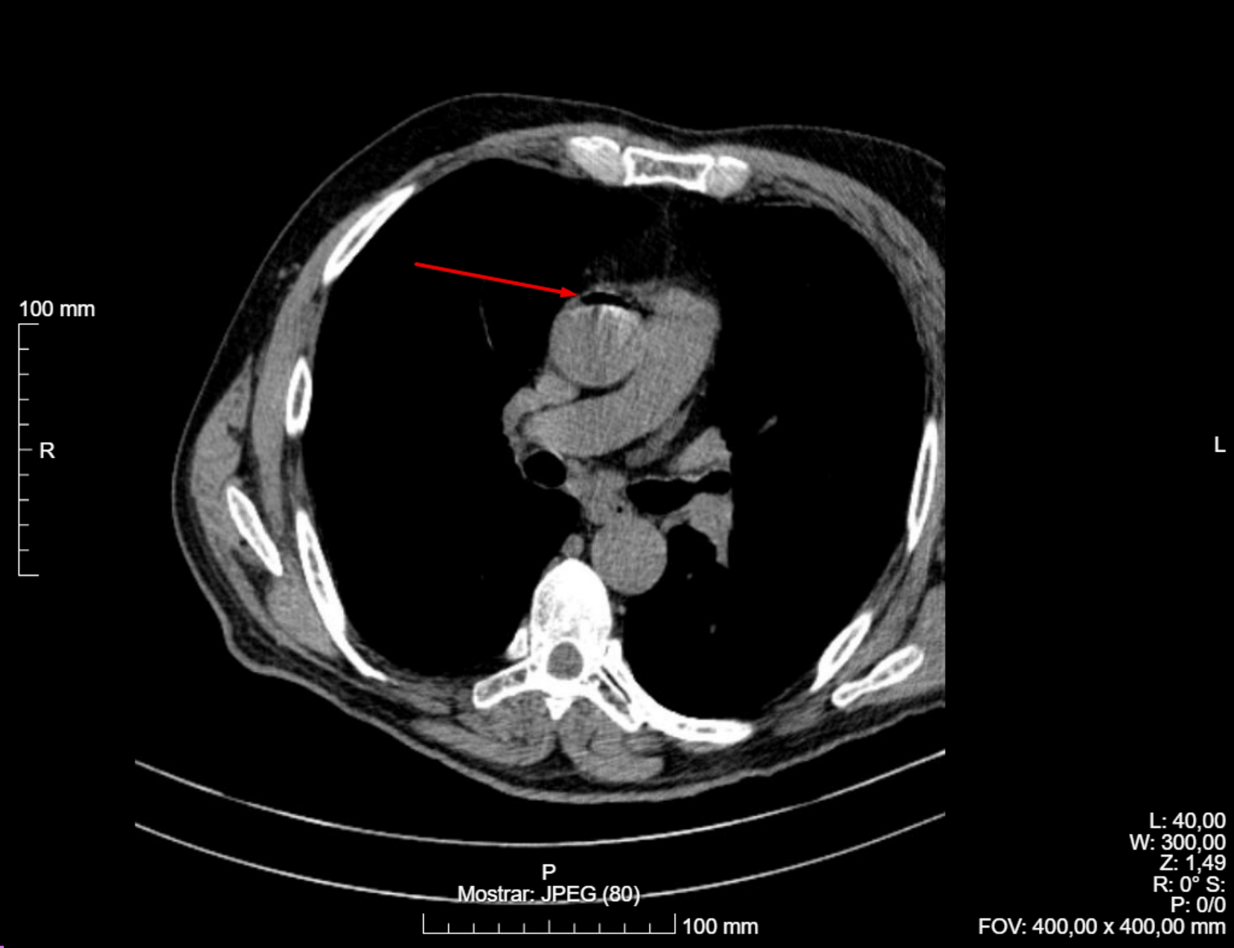
Control CT scan showing vascular (aorta) air embolism.

A post-biopsy control CT scan revealed the presence of air within the left ventricle and ascending aorta, consistent with an air embolism, as shown in Figures 1-2 (red arrow points to air embolism). Additionally, a minimal pneumothorax was observed, which did not require drainage, along with a small hemorrhage along the needle tract.

The patient was successfully resuscitated and intubated for mechanical ventilation and admitted to an intensive care unit. In the post-resuscitation state, the patient was ventilated in pressure-regulated volume control mode with a tidal volume of 500 mL, a respiratory rate of 24 cycles per minute, a positive end-expiratory pressure (PEEP) of 5 cmH_2_O, and an FiO_2_ of 60%, requiring minimal amine support after an analgesia bolus for procedures and consequent hypotension. The patient was sedated with 1% propofol via the peripheral route, maintaining a Richmond Agitation-Sedation Scale (RASS) score of -2. At admission, the patient had an Acute Physiology and Chronic Health Evaluation II (APACHE II) score of 15 and a Simplified Acute Physiology Score II (SAPS II) score of 34.

During the stay in intensive care, the patient was treated with inhaled ipratropium bromide at a dose of 20 mcg with four inhalations every six hours, inhaled beclometasone (250 mcg every eight hours), intravenous pantoprazole (40 mg per day), prophylaxis with subcutaneous enoxaparin sodium (40 mg), and alprazolam (0.5 mg per day), without the need for vasopressor support.

The patient was initially referred for hyperbaric oxygen therapy, but this option was not considered due to the presence of pneumothorax, a condition regarded as a contraindication. Additionally, there was no room for drain placement as the pneumothorax was very small. Later, he was transferred to the pulmonology unit and discharged home in a stable condition, fully recovered from this incident without any sequelae.

## Discussion

Air embolism is a rare but potentially fatal complication associated with various medical procedures, including laparoscopic surgeries, central venous catheterization, pacemaker placement, and, as described in this case, image-guided lung biopsy. The case study highlights the need for increased awareness among clinicians regarding the risk factors, early recognition, and prompt management of this condition.

During a lung biopsy, air can inadvertently enter the pulmonary venous system, particularly if the biopsy needle disrupts a pulmonary vein while the patient is inhaling. The presence of negative intrathoracic pressure can facilitate air entry, leading to an embolism that can travel through the left atrium and ventricle, ultimately reaching systemic circulation. If significant, this can result in cerebral, coronary, or other vital organ ischemia due to the embolism [7]. One of the risk factors for air embolism during lung biopsy includes biopsy of lesions located in the lower lung lobes where gravity may increase venous air entry, like in this case [8].

The clinical manifestations of air embolism can vary depending on the volume of air introduced and the vascular system involved. Symptoms may range from transient neurological deficits, dyspnea, and chest pain to cardiovascular collapse, as observed in this case.

A high index of suspicion is necessary, particularly in patients undergoing high-risk procedures. Imaging modalities such as CT can identify intracardiac or pulmonary air, while echocardiography can assist in detecting air bubbles in the heart chambers. Preventive strategies should be emphasized to minimize the risk of air embolism. These include using smaller gauge needles, performing biopsies in the expiratory phase, avoiding unnecessary multiple passes, and closely monitoring patients for early signs of embolism. Also, performing a transthoracic biopsy with the patient positioned ipsilaterally dependent, ensuring the lesion is below the level of the left atrium, seems to be an effective strategy for preventing systemic air embolism [9].

Early recognition and rapid intervention can improve the outcomes. The treatment includes immediate oxygen therapy with high-flow oxygen or hyperbaric oxygen therapy, which helps reduce the volume of the air embolism and mitigates ischemic damage. Patient positioning in the left lateral decubitus or Trendelenburg position may prevent air from entering the systemic circulation and facilitate its resorption in the right heart chambers. In cases of cardiovascular collapse, hemodynamic support, including fluid resuscitation, inotropic support, and cardiopulmonary resuscitation (CPR), may be required. Additionally, in select cases, aspiration of air through a central venous catheter or cardiac puncture has been reported as a potentially life-saving measure [10,11].

## Conclusions

CT-guided transthoracic needle biopsy is a valuable and commonly utilized technique for assessing pulmonary nodules. Although rare, cardiac air embolism is a potentially fatal complication of this procedure. Awareness of its pathophysiology, risk factors, and early management is critical for improving patient outcomes. Its life-threatening nature necessitates early detection and intervention, requiring careful clinical judgment which was done in this case. Healthcare providers should remain vigilant about the associated risk factors and recognize the typical signs and symptoms when performing this procedure as timely and appropriate intervention can be life-saving. Clinicians must maintain vigilance when performing high-risk procedures and adopt preventive strategies to mitigate the occurrence of this life-threatening condition. Continued research and reporting of similar cases will further enhance understanding and guide best practices in interventional pulmonology and radiology.
